# Weight Loss Disparities Among Hispanic and Underserved Participants, Colorado, 2015–2018

**DOI:** 10.5888/pcd17.200228

**Published:** 2020-12-24

**Authors:** Morgan N. Clennin, Allison Maytag, Jennifer Ellis, Andrea Wagner, Becky DiOrio, Cheryl Kelly

**Affiliations:** 1 Kaiser Permanente Colorado, Institute for Health Research, Aurora, Colorado; 2 Colorado Department of Public Health and Environment, Denver, Colorado

## Abstract

**Introduction:**

The National Diabetes Prevention Program (DPP) is an evidence-based strategy to prevent the development of type 2 diabetes in adults at high risk through education and behavior modifications that promote weight loss. This evaluation aimed to determine if National DPP participants’ weight-related outcomes varied across demographic subgroups, including sex, age, race/ethnicity, and insurance status, after controlling for program attendance and physical activity.

**Methods:**

Our cross-site evaluation used participant-level data from 11 organizations during July 2015 through June 2018. A modified Poisson regression model was used to examine the relationship between demographic subgroups, controlling for physical activity (minutes per week) and program attendance.

**Results:**

A total of 1,007 National DPP participants were included in the analyzed sample. Participants lost an average of 4% of their initial body weight, approximately 8 pounds. About one-third of participants achieved greater than 5% weight loss. In the unadjusted estimates, participants who were Hispanic, non-Hispanic Black, young, and uninsured were significantly less likely to achieve 5% or greater weight loss. Demographic differences in achieving 5% or greater weight loss, however, were not significant after adjusting for program attendance and physical activity level.

**Conclusions:**

Disparities in National DPP weight-related outcomes were not observed across demographic groups after adjusting for program attendance and physical activity levels. However, non-Hispanic Black participants had lower attendance and Hispanic participants reported less physical activity than participants of other races/ethnicities. Strategies to improve National DPP participation and increase physical activity, therefore, should be prioritized among Hispanic and non-Hispanic Black participants.

**Summary**

**What is already known on this topic?**

The National Diabetes Prevention Program (DPP) is an evidence-based strategy promoted by the Centers for Disease Control and Prevention to prevent the development of type 2 diabetes among adults at high risk.

**What is added by this report?**

Our evaluation provides a practice-based demonstration of the effectiveness of the National DPP in real-world settings among medically underserved racial/ethnic populations.

**What are the implications for public health practice?**

Findings showed non-Hispanic Black participants had lower attendance at National DPP sessions, and Hispanic participants reported lower physical activity levels. Strategies to improve National DPP participation and increase physical activity among Hispanic and non-Hispanic Black participants should be prioritized.

## Introduction

About 88 million people aged 18 years or older (34% of US adults) are estimated to have prediabetes (1), a condition characterized by elevated glycated hemoglobin A_1c_ (HbA_1c)_, or blood glucose levels that are higher than normal but do not meet the threshold for diabetes (2). People with prediabetes have an increased risk for developing type 2 diabetes and subsequently experiencing numerous adverse health consequences (3). Lifestyle modification is more effective in preventing or delaying the onset of diabetes compared with prescription medication intervention (4,5). Hence, public health efforts to develop evidence-based interventions promoting lifestyle modifications to prevent or delay the onset of diabetes have been prioritized over the past decade.

One promising lifestyle intervention for people with prediabetes is the National Diabetes Prevention Program (DPP). In 2012, the Centers for Disease Control and Prevention (CDC) selected the program for widespread implementation across the United States (6). The National DPP is an evidence-based intervention that emphasizes lifestyle modifications, such as increased physical activity, healthy diets, and sustained weight loss, to reduce risk for developing type 2 diabetes (7,8).

Extensive research has concluded that the National DPP is an effective lifestyle modification program (5,9,10); however, a need for practice-based evidence still exists to demonstrate that widespread implementation and dissemination of the National DPP is effective in real-world settings and among underrepresented populations. Literature remains mixed regarding National DPP effectiveness among low-income and racial/ethnic minority groups (11–13). Further evaluation of organization-led National DPPs delivered across diverse settings that focus on racially/ethnically diverse populations is needed to fill gaps in existing knowledge. Our primary objective in this cross-site evaluation was to determine if National DPP participants’ weight-related outcomes varied across demographic subgroups, including sex, age, race/ethnicity, and insurance type, after controlling for program attendance and physical activity.

## Methods

Data for the evaluation were part of a larger cross-site evaluation of the Colorado Department of Public Health and Environment’s (CDPHE) grant programs that fund organizations for prevention, early detection, and treatment of cancer, cardiovascular disease, and chronic pulmonary disease. Over 3 years, from July 2015 through June 2018, the funder selected 11 organizations (eg, local public health departments, nonprofit community-based organizations) to deliver the National DPP to people with diagnosed prediabetes. Each organization was expected to 1) develop infrastructure to implement the CDC-recognized National DPP, 2) enroll high-risk people in the National DPP by establishing partnerships with health care providers to develop referral systems and/or conduct outreach to populations with high-risk, 3) deliver the National DPP per CDC standards to achieve and/or maintain accreditation (ie, fidelity), 4) provide feedback to referring health care providers, and 5) work toward program sustainability. As part of the funding criteria, priority was given to organizations targeting disproportionately affected and medically underserved populations to reduce health disparities.

The Partners in Evaluation and Research (PiER) Center was contracted as an external evaluator of the CDPHE grants program. The PiER Center did not play a role in funding decisions or implementation of the National DPP. The PiER Center led the evaluation of the National DPP in collaboration with the funder. The institutional review board of Kaiser Permanente of Colorado’s Institute for Health Research approved the evaluation.

During the funding period, the 11 organizations offered 242 National DPP cohorts (ie, unique programs) in 110 community sites across 15 counties. The National DPP is a year-long accredited program for adults at high risk for developing type 2 diabetes (8). During months 1 through 6, at least 16 weekly sessions focused on physical activity promotion and weight loss. During months 7 through 12, participants engaged in a minimum of 6 monthly sessions focused on maintenance of lifestyle changes and weight loss (8,14). National DPP goals included achieving a 5% to 7% weight loss and engaging in 150 minutes of physical activity per week (6,8).

The PiER Center received deidentified participant-level data for 2,770 adults enrolled in the National DPP during July 2015 through June 2018 across 11 organizations. Eligibility criteria for inclusion in the analytic sample were 1) enrollment in National DPP during the funding cycle, 2) opportunity for a 12-month follow up from first session attended, 3) having a record of prediabetes determination documented before enrollment according to Diabetes Prevention Recognition Program (DPRP) standards, 4) documentation of 2 or more weight measurements and 1 or more physical activity measurement taken during program participation, 5) complete data for demographic variables of interest, and 6) attending 4 or more program sessions to meet attendance thresholds. Participants who did not meet the criteria were not included in the analytic sample. These criteria align with those in previous studies (8,14). Additionally, participants who were pregnant were excluded because weight loss during pregnancy was not appropriate in our study. A total of 1,763 participants were excluded from the sample to yield a final analytic sample of 1,007 participants (Figure). Compared with participants in the analytic sample, participants excluded from the analysis were more often Hispanic, female, aged 18 to 44 years, and either uninsured or with unknown health insurance status and had a higher average weight at baseline.

**Figure:**
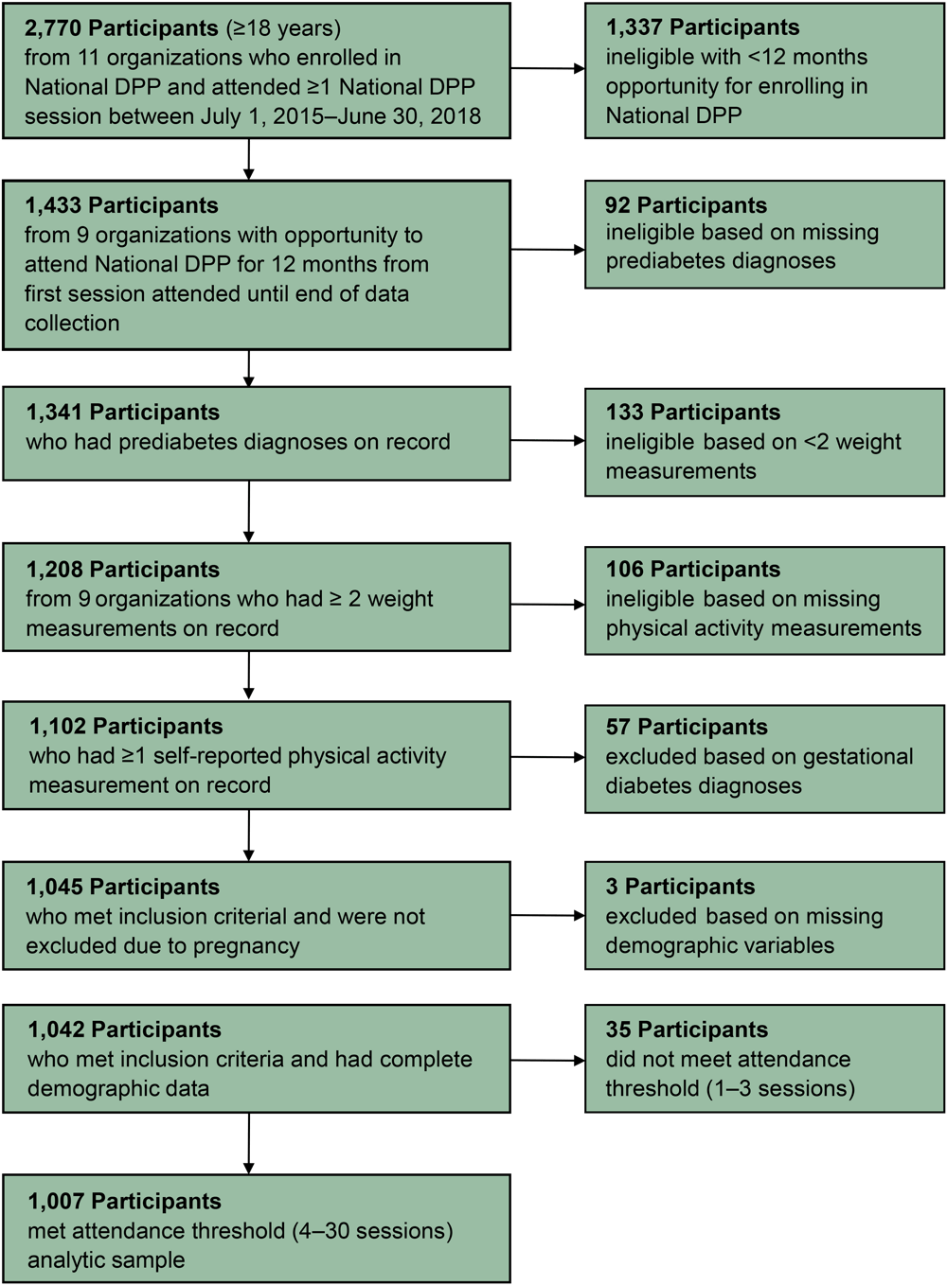
Flowchart for participant inclusion in National Diabetes Prevention Program (DPP) evaluation of weight loss disparities among racial/ethnic minority and underserved participants, Colorado, 2015–2018.

Demographic characteristics studied were sex, age, race/ethnicity, and insurance status. Sex was expressed as male or female. Age at enrollment was categorized as 18 to 44 years, 45 to 64 years, and 65 or more years; race/ethnicity as Hispanic, non-Hispanic Black, non-Hispanic White, other race/ethnicity (included multiracial), and unknown race/ethnicity. Insurance types were Medicaid, Medicare, private insurance, uninsured, other insurance, and unknown/missing insurance. Participant weights were measured during each session attended, per DPRP standards, and expressed in pounds (8). Weight measurements were used to determine percentage weight change from baseline (ie, weight at first measurement) to the follow-up (ie, last available measurement) by using the calculation first weight – last weight / first weight × 100. Using participants’ percentage weight loss, achievement of the DPRP standard was expressed as having 5% or more weight loss. Physical activity was self-reported by participants using a weekly activity tracker. During the week, participants recorded each instance of moderate-to-vigorous physical activity (MVPA), which could include activity across all domains (ie, leisure or recreational, domestic [eg, household chores], active transportation [eg, walking or biking to a destination], and occupational). The activity tracker was collected during each session, and total MVPA minutes per week was calculated. Average MVPA minutes per week was calculated as the sum of self-reported minutes of MVPA across all sessions attended divided by the number of sessions in which it was recorded. Meeting physical activity guidelines was determined as 150 minutes or more per week of MVPA (on average). Number of sessions attended was used to measure National DPP participant exposure, ranging from 4 to 22 sessions. Attendance was categorized into 3 groups according to level of participation (8): 1) high attendance (core sessions and maintenance) — participants attending 9 or more sessions in the first 6 months and at least 3 sessions in the second 6 months, 2) moderate attendance (core sessions only) — participants attending 9 or more sessions in the first 6 months but less than 3 sessions in the second 6 months, and 3) low attendance — participants who attended 8 or fewer sessions in the first 6 months, regardless of attendance in the second 6 months. Duration of National DPP exposure was expressed as number of months in the program and calculated as the number of days from the first to last session, divided by 30.4 and rounded (eg, results <1 month were rounded to 1 month). Details about National DPP measurements have been published (8).

Descriptive statistics were used to examine baseline characteristics and program outcomes for the overall sample and achievement of 5% or more weight loss. Differences were compared by using χ^2^ tests of proportion (categorical variables) or 2-sample *t* tests or Kruskal–Wallis tests (continuous variables). Weight loss is the primary outcome for the program. Next, a modified Poisson regression model with robust error variance using SAS GENMOD (version 9.4, SAS Institute Inc) was used to examine the relative risk (RR) for achieving a 5% or more weight loss (15). Because achieving a weight loss of 5% or more is relatively common, we estimated the RR for this outcome rather than the odds ratio (16). Covariates were sex, age group, race/ethnicity, insurance type, meeting physical activity guidelines, and attendance group. All models controlled for organization, initial weight, and duration of National DPP exposure. *P* values less than .05 were considered significant. Analyses were conducted by using SAS.

## Results

Of the 1,007 participants in the analyzed sample, 815 (81%) were female; mean age was 55.8 years (Table 1). The sample was racially and ethnically diverse with 46% non-Hispanic White, 39% Hispanic, 6% non-Hispanic Black, and 9% of other races/ethnicities, including multiracial and unknown. Medicare or Medicaid insured about 30% of sample participants, 25% had private health insurance, and 10% were uninsured. Relative to 5% or more weight loss versus less than 5% weight loss, no significant difference was observed by initial weight or sex. However, the percentage of participants who achieved 5% or more weight loss was significantly greater among older, non-Hispanic White, and Medicare-insured demographic groups compared with participants who did not achieve more than 5% weight loss.

**Table 1:** Characteristics of the National Diabetes Prevention Program (DPP) Study Group Overall and by Weight Change^a^, Colorado, 2015–2018

Characteristic	Total (N = 1,007)	<5% Weight Loss, (n = 668)	≥5% Weight Loss, (n = 339)	*P* Value^b^	*P* Value^c^
**Sex,** %					
Male	19.4	19.3	19.5	.95	NA
Female	80.6	80.7	80.5		
**Age**					
Age, mean (SD), y	55.8 (13.6)	54.3 (13.6)	58.7 (12.9)	<.001	NA
18–44 y, %	22.4	25.5	16.5	<.001	.001
45–64 y, %	47.8	48.7	46.0		.43
≥65 y, %	29.8	25.9	37.5		<.001
**Race/ethnicity,** %					
Hispanic	38.7	41.6	33.0	.004	.01
Non-Hispanic Black	6.4	7.2	4.7		.13
Non-Hispanic White	46.1	41.8	54.6		<.001
Other^d^	1.6	1.7	1.5		.84
Unknown	7.3	7.8	6.2		.36
**Type of insurance,** %					
Medicaid	11.8	13.5	8.6	.002	.02
Medicare	17.7	15.4	22.1		.01
Other	12.0	11.4	13.3		.38
Private	24.5	24.3	25.1		.77
Uninsured	10.3	12.3	6.5		.004
Unknown/missing	23.6	23.2	24.5		.65
Initial weight, mean (SD), lbs	196.4 (44.9)	197.0 (44.7)	195.3 (45.3)	.40	NA
**Initial BMI category,** %					
Underweight	0.2	0.2	0.3	.05^e^	1.00
Normal weight	7.7	8.1	6.8		.46
Overweight	29.2	26.2	35.1		.003
Obese	60.8	63.0	56.3		.04
Missing	2.2	2.5	1.5		.27

Abbreviation: BMI, body mass index; NA, not applicable.

a Values reported as column percentage or mean (SD), unless otherwise noted; percentages may not sum to 100% because of rounding to nearest decimal place.

b *P* value comparing proportions within groups.

c *P* value for post-hoc pairwise comparisons.

d Includes multiracial participants.

e Fisher exact test used.

About one-third (34%) of National DPP participants achieved 5% or greater weight loss over the course of their participation. These participants lost an average of 8 pounds from their initial weight to their last weight measurement, which is a mean of 4% weight change, averaging 0.25% per session attended (Table 2). National DPP participants attended an average 16 sessions (range, 4–30 sessions) and actively participated for almost 7 months. Approximately 8 of 10 participants attended 9 or more sessions during months 1 through 6 (ie, core sessions from the moderate and high attendance groups). Only 4 of 10 participants, however, completed the program according to the DPRP standard of attending 9 or more sessions during months 1 through 6 and 3 or more sessions during months 7 through 12 (ie, core sessions and maintenance from the high attendance group). The remaining 19% attended less than 8 National DPP sessions during months 1 through 6 (ie, the low attendance group). Participants who completed National DPP were more likely to be aged 65 or more years than younger, to have Medicare insurance than other insurance, and to be non-Hispanic White than Hispanic or non-Hispanic Black. Relative to physical activity, participants reported an average of 182 minutes per week of MVPA, and 52% reported meeting physical activity guidelines. Participants who achieved 5% or greater weight loss reported 57 minutes per week of MVPA more than those who did not (220 min/wk vs 163 min/wk, *P* < .001) and were more likely to meet physical activity guidelines (68% vs 44%, *P* < .001). Participants who met the physical activity threshold were more likely to be older than younger, male than female, and non-Hispanic White than other races/ethnicities, and to have Medicare insurance than other insurance.

**Table 2:** National Diabetes Prevention Program Participant Outcomes for Weight Loss, Physical Activity, and Program Attendance^a^, Colorado, 2015–2018

Outcome	Total (No. = 1,007)	<5% Weight Loss (n = 668)	≥5% Weight Loss (N = 339)	*P* Value^b^	*P* Value^c^
**Weight change**					
Weight change, mean (SD), lbs	8.0 (11.1)	2.6 (4.9)	18.7 (2.0)	<.001	NA
Weight change, %	4.1 (5.0)	1.3 (2.5)	9.4 (4.4)	<.001	
**Average physical activity, min/wk**					
Self-reported mean (SD)	181.9 (118.0)	162.5 (104.7)	220.0 (132.7)	<.001	NA
Percentage with ≥150	52.2	44.3	67.8	<.001	
**Attendance, mean, SD**					
No. of sessions attended	16.0 (6.4)	14.4 (6.2)	19.0 (5.6)	<.001	NA
No. months in program	6.9 (3.8)	6.0 (3.7)	8.6 (3.4)	<.001	
**Attendance groups^d^, %**					
Low attendance	19.3	25.3	7.4	<.001	<.001
Moderate attendance	40.2	44.8	31.3		<.001
High attendance	40.5	29.9	61.4		<.001

Abbreviation: NA, not applicable.

a Values are reported as column percentage or mean (SD) unless otherwise indicated. Percentages may not sum to 100% because of rounding to nearest decimal place.

b *P* value comparing proportions within groups.

c *P* value for post-hoc pairwise comparisons.

d Attendance groups based on Diabetes Prevention and Recognition Program standards (8). High attendance — core sessions and maintenance; participants attended ≥9 sessions in the first 6 months and ≥3 sessions in the second 6 months. Moderate attendance — core sessions only; participants attended ≥9 sessions in the first 6 months but <3 sessions in the second 6 months. Low attendance — participants attended ≤8 sessions in the first 6 months, regardless of attendance in the second 6 months.

In the unadjusted models, participants who were younger (18–44 y and 45–64 y), Hispanic, non-Hispanic Black, and uninsured were significantly less likely to achieve the weight loss goal compared with corresponding reference groups (Table 3). Additionally, participants who attended more sessions (ie, moderate attendance and high attendance) and participated in 150 minutes per week or more of MVPA were significantly more likely to lose at least 5% of their body weight.

**Table 3:** Relative Risk of Achieving Weight Loss of 5% or More Among National Diabetes Prevention Program Participants, Colorado, 2015–2018

Independent Variables	Unadjusted Relative Risk		Adjusted Relative Risk^a^	
	Relative Risk (95% CI)	*P* Value	Relative Risk (95% CI)	*P* Value
**Sex**				
Female	1 [Reference]		1 [Reference]	
Male	1.01 (0.81–1.25)	.95	1.03 (0.82–1.28)	.82
**Age, y**				
18–44	0.59 (0.45–0.76)	<.001	0.92 (0.68–1.25)	.60
45–64	0.77 (0.64–0.92)	.005	1.00 (0.82–1.22)	.99
≥65	1 [Reference]		1 [Reference]	
**Race/ethnicity**				
Non-Hispanic White	1 [Reference]		1 [Reference]	
Hispanic	0.72 (0.59–0.87)	<.001	1.10 (0.84–1.45)	.49
Non-Hispanic Black	0.63 (0.40–0.97)	.04	0.81 (0.51–1.27)	.36
Other	0.78 (0.38–1.64)	.52	0.91 (0.39–2.16)	.84
Unknown	0.72 (0.49–1.05)	.09	0.78 (0.57–1.08)	.14
**Insurance type**				
Private	1 [Reference]		1 [Reference]	
Medicaid	0.71 (0.49–1.02)	.06	0.81 (0.56–1.17)	.25
Medicare	1.22 (0.96–1.56)	.10	1.04 (0.80–1.36)	.75
Other	1.08 (0.81–1.44)	.60	0.99 (0.75–1.30)	.93
Uninsured	0.61 (0.41–0.93)	.02	0.76 (0.50–1.16)	.20
Unknown/missing	1.01 (0.79–1.29)	.92	0.84 (0.62–1.16)	.29
**Attendance groups** b				
Low	1 [Reference]		1 [Reference]	
Moderate	2.03 (1.36–3.03)	<.001	1.82 (1.14–2.90)	.01
High	3.96 (2.71–5.77)	<.001	2.15 (1.12–4.12)	.02
**Average physical activity, min/wk**				
<150	1 [Reference]		1 [Reference]	
≥150	1.93 (1.59–2.34)	<.001	1.56 (1.24–1.95)	<.001

a Adjusted model controlled for months in program, initial weight, and organization.

b Attendance groups based on Diabetes Prevention and Recognition Program standards (8). High attendance — core sessions and maintenance; participants attended ≥9 sessions in the first 6 months and ≥3 sessions in the second 6 months. Moderate attendance —core sessions only; participants attended ≥9 sessions in the first 6 months but <3 sessions in the second 6 months. Low attendance — participants attended ≤8 sessions in the first 6 months, regardless of attendance in the second 6 months.

In the fully adjusted models, age, race/ethnicity, and insurance were no longer significantly associated with achieving 5% or greater weight loss (Table 3). Attendance group (level of completion) and physical activity, however, were significantly associated with losing at least 5% of weight after adjusting for sex, age, race/ethnicity, insurance type, organization, attendance group and initial weight. Specifically, participants who met physical activity guidelines (≥150 MVPA min/wk) were nearly 60% more likely to achieve 5% or more weight loss compared with those who did not meet physical activity guidelines (RR, 1.56; 95% CI, 1.24–1.95). Compared with participants who attended less than 9 sessions (ie, low attendance group), participants with moderate and high attendance were 1.8 (95% CI, 1.14–2.90) and 2.2 (95% CI, 1.12–4.12) times more likely to achieve 5% or greater weight loss. Supplemental analyses revealed that Hispanic and non-Hispanic Black participants were significantly less likely to complete the program. Specifically, non-Hispanic Black participants consistently attended fewer sessions, although Hispanic participants attended significantly more sessions during the weight loss portion of the program but were still less likely to complete the program according to DPRP criteria. Additionally, Hispanic participants consistently reported significantly less physical activity and were less likely to achieve the recommended amount of physical activity.

## Discussion

The primary finding of our cross-site evaluation was that weight loss among a diverse sample of National DPP participants was not significantly different across sex, age, race/ethnicity, and insurance type after accounting for program attendance and physical activity. Previous studies hypothesized that low-income and medically underserved participants would be less likely to achieve the program goal of 5% or greater weight loss; however, our findings do not align with those studies that demonstrated that low income and medically underserved participants achieve significantly less weight loss compared with higher-income participants (12,14,17). For instance, Ritchie et al (17) found that 26.1% of non-Hispanic White participants achieved at least 5% weight loss compared to 15.6% of Hispanic participants, and Ely et al (14) found non-Hispanic White participants had an adjusted percentage weight loss of 4.6% more compared with non-Hispanic Black participants, who had an adjusted weight loss of 3.2%. One explanation for this discrepancy in findings might be that our evaluation controlled for program attendance and physical activity. Another possible explanation is that our evaluation included a large sample of participants who were more racially/ethnically diverse compared with those in other studies.

Our evaluation did observe disparities in weight loss across race/ethnicity groups in the unadjusted models. Specifically, participants who were Hispanic and non-Hispanic Black were less likely to achieve the weight loss goal compared with non-Hispanic White participants. These differences dissipated after controlling for attendance group and physical activity level, suggesting these factors are likely the primary drivers of weight loss among National DPP participants. Notably, differences in physical activity engagement and attendance group were observed across race/ethnicity groups in supplemental analyses. Specifically, non-Hispanic Black and Hispanic participants consistently attended fewer sessions and Hispanic participants were less likely to achieve the recommended amount of physical activity. These findings suggest that 1) facilitators and barriers to program attendance or completion may differ among non-Hispanic Black and Hispanic participants, and 2) current efforts to promote physical activity among Hispanic participants may not be adequate to increase activity levels.

Program retention among non-Hispanic Black participants and Hispanic Black participants has been documented to be lower compared with non-Hispanic White participants (18,19). Strategies to promote retention, however, have not been rigorously evaluated or published in peer-reviewed literature. Several factors probably contribute to lower attendance among groups at higher risk, such as limited access to social and economic resources (eg, child care, transportation, flexible work schedules). Future evaluations are needed to examine promising strategies that will address participation barriers among non-Hispanic Black and Hispanic participants. Among Hispanic populations, lower physical activity levels have been well documented (14,20). Some factors attributed to the disparity include cultural differences, access to safe or affordable places to engage in physical activity, and lack of time or other resources to engage in physical activity (21–23). To improve National DPP effectiveness among Hispanic participants, strategies to promote physical activity should be prioritized. Other studies suggest that tailoring to community-specific needs and cultural norms might increase effectiveness in certain racial/ethnic groups (24,25). For example, CDC’s Prevent T2 (type 2 diabetes) curriculum was developed independently and incorporates culturally appropriate examples of physical activity for Hispanic communities (26). Although this is a culturally tailored curriculum, broader adoption and delivery in Spanish among organizations serving predominately Hispanic communities is needed to improve program effectiveness. Organizations serving Hispanic communities should also consider the location of programming and provide services in safe community spaces, led by trusted sources. Another promising strategy is the provision of transportation to activities, such as recreation centers and swimming pools to increase safe and accessible opportunities for physical activity (27). Additionally, using community health workers (*promotores de salud)* and lifestyle coaches to provide program support, such as creating buddy programs or establishing walking groups, might be effective at increasing physical activity (27). Finally, organizations might consider additional CDC-approved and adapted curricula that better align with the community being served. More evaluation is needed to assess the effectiveness of these strategies to increase physical activity among Hispanic participants in the National DPP.

Our evaluation contributes to the existing body of literature and addresses several gaps in the knowledge base. This study is one of the few to conduct a cross-site evaluation of National DPP programs delivered across diverse communities, organizations, and settings (10,14). Unlike other studies (10,14), our analytic approach was more comprehensive, shedding light on previously reported disparities. We were able to examine National DPP effectiveness across demographic characteristics by using a large and racially/ethnically diverse sample. Despite these strengths, some limitations should be acknowledged. First, our evaluation was conducted in a real-world setting that lacked a control group. Although the real-world setting can be a strength, selected organizations may not have been represented, which might have influenced findings. Second, program staff conducted program implementation and outcome measurements. Although all organizations were accredited to deliver the National DPP, program delivery, outcome measurement, and outcome reporting might have varied across organizations. Unfortunately, the evaluation team was not able to assess variations in implementation across organizations. In addition, physical activity was self-reported. This may have resulted in overestimation or underestimation of activity levels, although all participants received instruction regarding physical activity reporting. Finally, our evaluation was limited to the data collected through the grant program and did not include information that might have shed light on the observed findings (eg, barriers and facilitators to success).

Our cross-site evaluation observed that approximately 34% of National DPP participants achieved more than 5% weight loss. Previous research has shown that the National DPP effectively reduces participant body weight and risk for developing type 2 diabetes (4,5,10,14). Translation of this program to real-world settings and delivery to medically underserved populations and those at increased risk, however, may yield less effective outcomes. In alignment with previous studies (14,17,28), our findings highlight that program attendance and engaging in the recommended amounts of physical activity are key drivers of program effectiveness. Not all participants, however, are able to meet attendance and physical activity recommendations. Our evaluation indicated that Hispanic participants might experience disparities in weight loss because of consistently lower engagement in physical activity, and non-Hispanic Black participants may experience disparities in weight loss because of lower attendance in the National DPP. Efforts are needed to promote physical activity among Hispanic participants and engagement among non-Hispanic Black participants. Additionally, future research is needed to better understand facilitators and barriers to achieving weight loss goals among Hispanic and non-Hispanic Black populations.
